# Potential Role of Chemokines in Fracture Repair

**DOI:** 10.3389/fendo.2017.00039

**Published:** 2017-03-02

**Authors:** Bouchra Edderkaoui

**Affiliations:** ^1^Musculoskeletal Disease Center, Loma Linda VA Health Care Systems, Loma Linda, CA, USA; ^2^Department of Medicine, Loma Linda University, Loma Linda, CA, USA

**Keywords:** fractures, bone, inflammation, chemokines, cell migration, delay fracture repair

## Abstract

Chemokines are a family of small cytokines that share a typical key structure that is stabilized by disulfide bonds between the cysteine residues at the NH_2_-terminal of the protein, and they are secreted by a great variety of cells in several different conditions. Their function is directly dependent on their interactions with their receptors. Chemokines are involved in cell maturation and differentiation, infection, autoimmunity, cancer, and, in general, in any situation where immune components are involved. However, their role in postfracture inflammation and fracture healing is not yet well established. In this article, we will discuss the response of chemokines to bone fracture and their potential roles in postfracture inflammation and healing based on data from our studies and from other previously published studies.

## Introduction

Bone fracture healing is a complex progression of events that require a timely sequence of interactions between cells and their mediators. Both resident and infiltrating cells contribute to the three phases of fracture healing: inflammation (initial hematoma with subsequent infiltration of inflammatory cells), bone formation, and bone remodeling (1, 2).

Approximately 7.9 million fractures occur in the United States every year, and nearly 5–20% exhibit delay or disruption in healing, resulting in significant morbidity and reduced productivity (3, 4). Thus, understanding the molecular mechanisms involved in postfracture inflammation and repair have the obvious potential to improve the quality and the time of fracture healing and translate into significant therapeutic benefit in both patient outcomes and reduced costs to society.

Delayed bone fracture healing and non-union fractures represent an important clinical problem, especially in patients with open fractures, patients with diabetes, and patients with multiple fractures who also suffer from posttraumatic systemic inflammation. However, the underlying biochemical and cellular mechanisms that become dysregulated during delayed union and non-union fracture repair remain controversial.

A key initial step in fracture repair is an inflammatory reaction involving immune cells that become activated immediately in response to tissue damage. Although much is known about the function of inflammatory cells as well as the other cells that migrate within the fracture in response to injury, little is known about the chemotactic and activation signals that influence this response. It is now well accepted that chemokines promote inflammation (5–7) and angiogenesis (8–10). In addition, chemokines are thought to play an important role in several aspects of bone metabolism including the recruitment of leukocytes and the formation of osteoclasts (11, 12). Therefore, they may contribute to the regulation of osteoneogenesis, by integrating inflammatory events and the reparative processes important in modulating fracture healing.

This review will discuss chemokine expression in response to bone fracture and the potential role of these molecules in postfracture inflammation and healing.

## Chemokines and Chemokine Receptors

Chemokines belong to a family of small cytokines. There are approximately 50 chemokines (13, 14) interacting with some 23 different receptors (15–19). They range in size from 8 to 20 kDa and share a basic structure that is stabilized by disulfide bonds between cysteine residues. Based on the pattern of cysteine residues near the *N*-terminus, chemokines can be divided into four subfamilies (20): (1) the CC subfamily, which includes beta chemokines, has cysteine residues adjacent to each other; (2) the CXC subfamily, which includes alpha chemokines, has cysteine residues that are separated by an intervening amino acid; (3) the C subfamily has one cysteine residue at the *N*-terminus of the protein; and (4) the CX3C family, with only one representative, CX3CL1 (also known as neurotactin and fractalkine), in which the two cysteine residues are separated by three amino acids (21).

Chemokines are secreted by a great variety of cells such as mononuclear leukocytes (22–24), neutrophils (25–28), eosinophils (29), fibroblasts (30, 31), blood endothelial cells (32), and adipocytes (33, 34). They can be induced in several different conditions. The name “chemokine” is derived from the ability of chemokines to induce directed chemotaxis in nearby responsive cells. Some chemokines are considered pro-inflammatory and can be induced during an immune response to attract cells of the immune system to a site of infection, whereas others are considered homeostatic and are involved in controlling the migration of cells during normal physiological conditions (35, 36). Chemokines play roles in cell maturation and differentiation, infection, autoimmunity, cancer, and, in general, in any situation where immune components are involved. However, their role in postfracture inflammation and fracture healing is not yet well established.

Chemokine function is directly dependent on the interaction with chemokine receptors. Chemokine receptors are G protein-linked transmembrane receptors located on the surfaces of target cells (15, 37). Chemokines bind their cognate G protein-coupled receptors and trigger intracellular calcium fluxes. As a concentration of chemokines increases, calcium flux signaling continue to increase till it reaches a plateau, while cell migration increases with the increase of chemokine concentration but then returns to baseline. Typically, a moderate increase in chemokines’ concentration leads to chemotactic migration, but a huge increase in the concentration of chemokines could halt cell migration (38). However, the signaling mechanisms that govern this phenomenon remain unclear.

There are 20 signaling chemokine receptors (37) and three non-signaling or scavenger receptors that serve to mediate chemokine-dependent signaling by binding, internalizing, and degrading chemokines (39, 40). Though similar to other seven-transmembrane receptors, signaling chemokine receptors share certain structural features, such as the highly conserved DRYLAIV amino acid sequence in the second intracellular loop (41), a feature that is absent in decoy receptors, indicating its implication in signaling. Several studies have shown that chemokine receptors are expressed on the surface of cells as both homo- and heterodimers (42–45), but the stability of the dimer is likely dependent on the presence of the ligand.

In a recent article, Muñoz et al. (46) have described the different types of dimerizations that occur between chemokine receptors, with ligands and receptors forming complexes in a dynamic equilibrium (47, 48), underscoring the complexity of chemokine activities. However, the lack of specific tools for stabilizing heterodimeric complexes, combined with the constantly changing equilibrium between receptor conformations, has complicated the studies of signaling functions of each dimer and limited our ability to modulate chemokine/receptor interaction using pharmacological approaches.

## Changes in the Expression of Chemokines in Response to Fracture and Their Potential Role in Fracture Healing

Although the function of the various cell types involved in postfracture inflammation is well established, the molecular mechanisms underlying the different phases of the bone repair process are still poorly understood. A thorough elucidation of how the spatial and temporal expression of chemokines and their receptors are modulated postfracture is ultimately essential to our understanding of the role of these chemokines in fracture repair.

Neutrophils are one of the most important cell types in the postfracture inflammation response. They are the first cell type to arrive at the fracture site in response to injury (49). They also express and produce chemokines that serve to attract further immune cells that ultimately participate in the healing process. A recent study, making use of a rat model in which a 5-mm bone defect was created in the femur, has reported the detection of neutrophils, a few granulocytes, and a few monocytes at the site of the defect as early as 12 h postfracture (49). The study also found that the number of neutrophils peaked at 24–48 h postfracture, marking the beginning of the inflammatory stage in this particular model system. Eosinophils and basophile granulocytes were seen 24 h postsurgery. In addition, protein expression of the major pro-inflammatory cytokine IL-6 and three major neutrophil chemoattractants, CXCL1, CXCL2, and CXCL3 (49), showed an increase in concentration immediately after surgery and a re-equilibration to baseline before 24 h postsurgery.

Another chemokine reported to play a role in neutrophil migration is monocyte chemotactic protein (MCP) 1, a member of CC chemokine family. MCP-1 has also been referred to as CCL2. It is a ligand for CCR2 but can also bind the Duffy antigen receptor for chemokines (DARC) (50). CCL2 is one of the first and most highly expressed chemokines in response to fracture in both animal models (51–54) and human fractures (55) (Table 1). CCL2 is involved in regulating neutrophil migration (56), angiogenesis, and macrophage infiltration in several inflammatory processes (57, 58) as well as regulating the migration of CD4+ T regulatory cells (59). Furthermore, CCL2 has been shown to be expressed at the periosteum around the fracture site during fracture healing (53), suggesting that CCL2 is involved in both postfracture inflammation and bone remodeling.

**Table 1:** **Chemokines that are upregulated during acute phase of fracture healing**.

Chemokine name	Specific chemokine receptors	Type of fracture (fracture model bone animal model)	Reference
CCL2 [monocyte chemotactic protein (MCP) 1]	CCR2	Stress fracture (axial loading—ulna—rat)	Wu et al. (51) Rundle et al. (52) Xing et al. (54) Ishikawa et al. (53) Hoff et al. (55)
		Single fracture (3-point bending—tibia—mouse)	
		Not stabilized single fracture (3-point bending—tibia—mouse)	
		Single fracture (rib - mouse)	
		Single fracture (human)^a^	

CCL3 [macrophage inflammatory protein 1 alpha (MIP-1a)]	CCR1, CCR5	Single fracture (3-point bending—tibia—mouse) Single fracture (human)^a^	Rundle et al. (52), Hoff et al. (55)

CCL4 [macrophage inflammatory protein 1 beta (MIP-1b)]	CCR1, CCR4, CCR5	Single fracture (femur-human)^a^	Hoff et al. (55)

CCL5 (Regulated upon Activation, Normally T-Expressed, and presumably Secreted)	CCR1, CCR3, CCR5	Single fracture (femur-human)^a^	Hoff et al. (55)

CCL7 (MCP-3)	CCR1, CCR2, CCR3	Not stabilized single fracture (3-point bending—tibia—mouse). Single fracture (Femur–human)^a^	Xing et al. (54), Hoff et al. (55)

CCL8 (MCP-2)	CCR1, CCR2, CCR5	Not stabilized single fracture (3-point bending—tibia—mouse)	Xing et al. (54)

CCL11 (Eotaxin)	CCR3, CCR2, CCR5	Single fracture (femur—human)^a^	Hoff et al. (55)

CXCL1, CXCL2, CXCL3	CXCR2	Segmental defect (5 mm bone defect—femur—rat)	Förster et al (49)

CXCL10 (IFN-γ-inducible protein 10)	CXCR3	Single fracture (femur—human)^a^	Hoff et al. (55)

CXCL8 (interleukin-8)	CXCR1, CXCR2, IL8R	Single fracture (femur-human)^a^	Hoff et al. (55)

CXCL12 (stroma cell-derived factor 1)	CXCR4, CXCR7	Segmental defect and live bone graft (mouse)	Kitaori et al. (90)

*^a^Fracture type not reported*.

It has been previously reported that tumor necrosis factor alpha (TNFα) enhances neutrophil recruitment in early postfracture inflammation and promotes the recruitment of monocytes by stimulating CCL2 production (7). Furthermore, depletion of neutrophils by Ly6G neutralizing antibody or inhibition of the CCL2 chemokine receptor CCR2 in a murine tibial fracture model resulted in significantly impaired fracture healing (7), while early treatment with TNFα increased neutrophil infiltration to the fracture area, as well as the expression of CCL2, when compared to untreated fractures (7). In another study with open tibial fracture, it has been reported that local treatment with low concentrations of TNFα enhanced fracture repair (60). This illustrates the importance of postfracture inflammation and especially the roles of TNFα and CCL2 in fracture healing. Moreover, the expression level of CCl2 was increased in patients with systemic skeletal disease such as osteoporotic patients (61), and the serum level of CCL2 was greater up to 4 weeks postsurgery in patients with type 2 diabetes compared to healthy patients (62). In both cases, patients suffered from fragility fractures, and the delay in fracture healing was obvious in type 2 diabetic patients, which suggested that increased level of CCL2 in the serum is one of the leading causes to impaired fracture healing in diabetic patients.

In an anabolic regimen, intermittent treatment with parathyroid hormone (PTH) caused MCP-1 expression to increase over time, eventually reaching 200-fold higher levels after 14 days of treatment. This in turn was accompanied by an increase in bone volume in the PTH-treated animals, compared to untreated control animals (63). Together, these data suggest that in addition to its role in chemotaxis of monocytes and neutrophils and osteolysis (64) during inflammatory bone remodeling, CCL2 might also be involved in bone formation during skeletal repair. However, this remains to be tested.

Macrophage inflammatory protein 1 alpha, also known as CCL3, binds to CCR1 and CCR5 (Table 1), which are receptors that mediate CCL3 chemotactic functions. CCL3 is produced by macrophages, natural killer cells, fibroblasts, and mast cells. Its expression in fracture callus was found to be upregulated during the first 3 days postfracture in both animal (52) and human models (55). In models with systemic inflammation, such as a rat diabetic model, increased levels of CCL3 in serum was also associated with delayed fracture healing, indicating the importance of a well-controlled inflammation on the overall process of fracture healing (65). In contrast to CCL2, the role of CCL3 in postfracture healing has not been well investigated.

Macrophage inflammatory protein 1 beta, also known as CCL4, binds the CCR1, CCR4, and CCR5 receptors (Table 1). CCL4 is secreted by major leukocytes such as T cells, B cells, and monocytes (66), and its expression was observed to be upregulated in fracture hematoma within 3 days postfracture, in parallel with an increase in the number of monocytes (55). Chondrocytes, in particular hypertrophic chondrocytes, have been identified as another source of CCL4. Expression of CCL4 by these cells has been shown to be dependent on TNFα in diabetic fractures (67). This suggests a role for TNFα and CCL4 in the loss of cartilage that is observed during the process of diabetic fracture healing. However, Lin et al. (68) have reported a differential expression of CCL4 in mesenchymal stem cells (MSCs) treated with high-mobility group box. These data together illustrate a complex role of CCL4 in fracture healing that still needs to be investigated.

The chemokine regulated upon Activation, Normally T-Expressed, and presumably secreted, also called CCL5, binds CCR1, CCR3, and CCR5 (Table 1) and is expressed in T lymphocytes. CCL5 promotes the recruitment and activation of inflammatory cells such as monocytes (69), lymphocytes (70), mast cells (71), and eosinophils (72). In the case of infection by pathogens, it is known to play a protective effect, through its interaction with CCR5 and the downstream ERK1/ERK2 and AKT signaling pathways. In a study of human fractures, CCL5 levels increased both at the site of the fracture hematoma and in the surrounding bone marrow (55). The magnitude of increase was greater in the fracture hematoma compared to surrounding bone marrow, an effect that can be likely explained by increased infiltration of T cells.

Monocyte chemotactic protein 3, also known as CCL7, binds the CCR1, CCR2, and CCR3 receptors. CCL7 is another mediator of pro-inflammatory pathways by virtue of its ability to activate leukocytes (73, 74). Previous studies have identified CCL7 as a homing factor for MSCs (75). In a mouse model, mRNA levels of CCL7 increased as early as 1 day postfracture (53), peaked at 2 days postfracture (53, 54), with a subsequent decline beginning at 3 days postfracture, but combined trauma model resulted in a significant increase in the level of CCL7 in plasma as early as 6 h postfracture that lasted up to 3 days postinjury compared to control non-injured animals (76).

Monocyte chemotactic protein 2, also known as CCL8, binds to the CCR1, CCR2, and CCR5 receptors. CCL8 attracts leukocytes and possesses various immunomodulating functions. Like other CC family chemokines, it influences mononuclear cell types (77). CCL8 was found to be upregulated (Table 1) during the first 7 days postfracture (54).

Eotaxin, also known as CCL11, binds the CCR2, CCR3, and CCR5 receptors, thus affecting the migration of eosinophils that express the CCR3 receptor, as well as monocytes that express both CCR2 and CCR5 (78). It has been reported that pretreatment of human monocytes with eotaxin reduces the binding of CCL2, the selective ligand for the CCR2, to monocytes (78) as well as the binding of CCL5 and CCL4 to CCR5. In previous studies (78), pretreatment of human monocytes with eotaxin triggered CCR5 activity at low concentrations of the ligand, while CCR2 was not activated by doses as high as 1 μM eotaxin, which suggested that eotaxin is a CCR5 agonist and a CCR2 antagonist (78). In fracture calluses, CCL11 was found to be upregulated within 3 days postfracture (Table 1) in human models (55).

IFN-γ-inducible protein 10 (IP10), also called CXCL10, binds to CXCR3 and is secreted from a variety of cells, including monocytes, endothelial cells, and fibroblasts, in response to interferon (79). CXCL10 inhibits bone marrow colony formation (80). It is a chemoattractant for human monocytes and T cells and promotes T cell adhesion to endothelia (80). Its expression is upregulated by both interferons and other inflammatory stimuli. It was found to be upregulated in fracture surrounding bone marrow in humans (Table 1), in parallel with an increase in the level of IFN-γ and TNFα and an increase in the number of CD3+ and CD3+CD4+ T cells in the surrounding bone marrow (55). Serum level of CXCL10 was found to be elevated in fracture patients with type 2 diabetes mellitus compared to patients with diabetes without fracture and normal patient with fracture (81). However, what role IP10 plays during fracture healing remains to be determined.

Interleukin-8, or CXCL8, binds the CXCR1, CXCR2, and IL-8R receptors (82, 83). IL-8 induces migration of hematopoietic progenitor cells through stimulation of the β2-integrin LFA-1 pathway (84). IL-8 levels have also been shown to be upregulated to a greater degree in fracture hematoma than in the surrounding bone marrow (Table 1), in parallel with an increase in the number of monocytes, granulocytes, and CD34+ HSCs (55). This suggests a role for IL-8 in HSC infiltration in response to bone injury.

Stroma cell-derived factor 1, also called CXCL12, binds the CXCR4 and CXCR7 receptors on the cell surface of responsive cells (85, 86). Local expression of CXCL12 has been shown to attract hematopoietic and endothelial progenitors to ischemic sites (87, 88). It is also expressed in bone marrow stroma cells (89) and has been reported to be upregulated at the endosteal surface around the injured bone from 7 to 14 days postsurgery (89). However, in other studies, CXCL12 levels have been reported to peak at different time points postfracture. For example, in a murine segmental bone graft model, CXCL12 levels were increased at the periosteum of the live bone graft from the first day of surgery, and its level continued increasing with time (90). In another murine model of fracture healing, CXCL12 expression was found in the fracture callus of hypertrophic cartilage and in immature cartilage near the pre-existing cortical bone (91). One explanation for the discrepancy in the time when CXCL12 expression peaks after fracture may be due to the specific nature of the injury. In some injuries, oxygen tension may change rapidly and since CXCL12 is reportedly regulated by a hypoxia-specific transcription factor, hypoxia-inducible factor 1, the expression of CXCL12 may increase rapidly after the blood supply is stopped in those models (92).

CXCL12 is well accepted as a major chemokine that plays a critical role in fracture repair. It is involved in fracture repair through possibly two mechanisms. One is by recruiting endothelial progenitor cells, thus contributing to increased angiogenesis (87), a key phase in fracture repair. The other involves enhancing the homing of osteoblastic progenitors to promote new bone formation (93).

In the presence of inflammation, endothelial cells are stimulated to increase the surface expression of adhesion molecules, such as selectins, as well as integrin ligands such as vascular cell adhesion molecule-1 and intercellular adhesion molecule-1. Subsequently, chemokines produced at the site of injury bind and activate chemokine receptors that are present at high concentrations on the surface of endothelial cells (94, 95). Once activated, chemokine receptors permit the transcytosis of chemokines from one side to other side of vascular endothelial membrane, resulting in chemotaxis. The level of chemokines and the time of their bioavailability around the injured bone and in blood circulation are key factors that influence cell recruitment to the injured bone for subsequent fracture repair.

## Functional Studies Using Knockout Mice

Animal models involving targeted knockout (KO) of selective chemokines and their receptors have been used to evaluate the role of chemokines in fracture repair process. Studies involving KO of DARC (52), CCL2/CCR2 (53), and CXCL12/CXCR4 (90) have illustrated a key role of chemokines in fracture healing. However, the role for a number of other chemokines such as CCL3 (96), CCL5 (97, 98), and CCL7 (75), found to be upregulated during the early phase of fracture healing and function as chemoattractants for MSCs, remains to be elucidated.

Postfracture inflammation has been evaluated in our mouse model using standard closed femoral fracture at the mid-shaft (52). The mRNA expression levels of IL-1β, IL-6, tumor necrosis factor, and CCL2, which binds to DARC and CCR2, were increased 1 day postfracture. However, the magnitude of increase was lower in DARC-KO fracture calluses, consistent with a reduced inflammatory response. Accordingly, the number of macrophages was significantly reduced around the fractures in DARC-KO mice compared to wild type mice. This was associated with greater collagen (COL) II expression at 3 days and COL-X at 7 days postfracture, compared to wild-type mice, suggesting that lack of DARC expression in DARC-KO mice led to an early or premature fracture cartilage formation and differentiation. However, by 21 days postfracture, histological analysis did not show any difference in fracture healing between DARC-KO and wild-type mice. This may have been the result of a reduction in the recruitment of osteoclast precursors to the fracture callus in DARC-KO mice, which in turn has increased the time required for the transition from cartilage callus to bone.

By using a rib fracture model and graft exchanges, Ishikawa et al. (53) have reported delayed fracture healing at 21 days postfracture in both CCL2-KO and CCR2-KO mice and that blockade of the CCR2 receptor only in the early phase of healing caused delayed fracture healing in wild-type mice. The discrepancy between our model and the CCL2-KO model could be due to CCL2 expression not being sufficiently reduced in DARC-KO mice so as to cause a delay in fracture healing (52). Furthermore, the finding that CCL2 exhibits a significant chemotactic effect on neutrophils (56) and MSCs (53) but has no effect on osteogenesis or chondrogenesis (53) suggests that the effect of CCL2 on fracture healing occurs *via* early neutrophil recruitment and MSC recruitment to the fracture site for subsequent bone formation.

Other studies have also reported the importance of MSC recruitment in fracture healing (99). By using heterozygous CXCL12+/– and CXCR4+/– mice, Kitaori et al. (90) have demonstrated that CXCL12 recruits MSCs to the injured bone postfracture for subsequent bone formation. Furthermore, it has been reported that following fracture, a CXCL12- and BMP2-positive perivascular cell population is recruited along the endosteum. This is then followed by an increase in BMP2 levels that leads to downregulation of CXCL12, a step that is essential for the differentiation of CXCL12 and BMP2+ cells during osteogenesis. Moreover, CXCL12 has been shown to regulate BMP-2-stimulated osteogenic differentiation (100), while the CXCR4 receptor is involved in regulating osteoblast development in postnatal bone (101). Therefore, we conclude that CXCL12 signaling may have roles in fracture healing that extend beyond cell recruitment, including direct effects on MSC proliferation and differentiation into cells of the chondrogenic and osteogenic lineages.

## Conclusion

**Figure 1:**
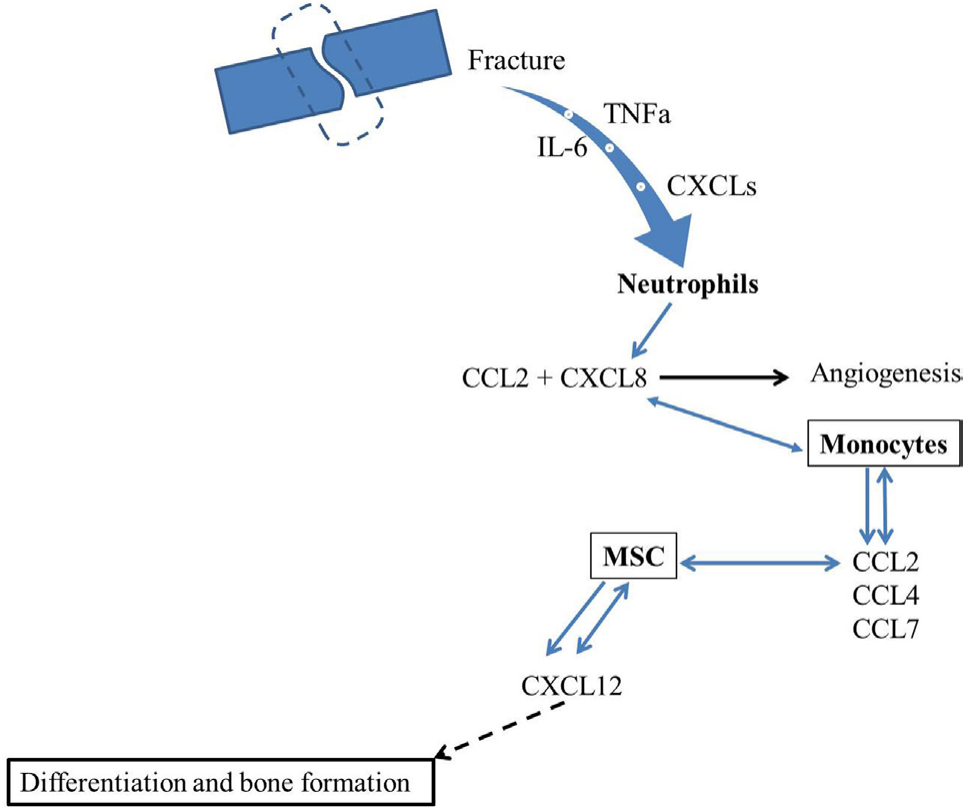
**Hypothetical model for the involvement of chemokines in fracture healing**. Fracture induces secretion of TNFα and IL-6 as well as CXCLs that attract neutrophils. Neutrophils will induce monocyte chemotaxis through CXCL8 and CCL2 secretion. Then, since monocytes secrete several chemokines, such as CCL2, CCL4, and CCL7, that are known to attract MSC. These later will migrate toward fracture callus and secrete CXCL12 that will bind to CXCR4 and regulate osteogenesis and fracture healing.

In conclusion, various chemokines are involved in postfracture inflammation and healing, and their induction and involvement in the whole process are dose, site, and time dependent. Two chemokines have been investigated extensively for their role in fracture healing: CCL2 and CXCL12. CCL2 is involved in neutrophil recruitment, which is an early stage of fracture healing and in MSC infiltration for subsequent fracture repair. The importance of CCL2 and its specific receptor CCR2 in the progress of fracture healing have been demonstrated in mouse models (KO mice) that lack CCL2 or CCR2 expression and which showed delayed healing. However, an increase in CCL2 levels in plasma postfracture has been associated with a likelihood of delayed fracture healing. CXCL12, which is expressed in bone marrow and perivascular stroma cells, is crucial for the recruitment of MSC to the injured bone postfracture, a necessary step for subsequent bone formation. On the basis of the above findings, we proposed a model (Figure 1) where fracture induces secretion of TNFα and Il-6, as well as CXCLs that attract neutrophils. Neutrophils would be expected to induce monocyte chemotaxis *via* stimulation of CCL2 secretion. Then, once monocytes secrete several chemokines, such as CCL2, CCL4, and CCL7, known to attract MSC, these cells will be induced to migrate toward fracture callus and secrete CXCL12. CXCL12 will in turn bind to CXCR4 and regulate BMP2 effects on osteogenesis and fracture healing. Currently, no published study has investigated the involvement of CCL4 and CCL7 in fracture healing. Thus, the issue of whether other chemokines are involved in fracture healing and how they interact with each other in the fracture healing process remains to be investigated. A comprehensive understanding of the role of chemokines in the fracture healing process could lead to development of chemokine-based therapies to promote healing of non-union fractures.

## Author Contributions

BE prepared the manuscript and collected the data.

## Conflict of Interest Statement

The author declares that the research was conducted in the absence of any commercial or financial relationships that could be construed as a potential conflict of interest.
